# Rumen microbiome in dairy calves fed copper and grape-pomace dietary supplementations: Composition and predicted functional profile

**DOI:** 10.1371/journal.pone.0205670

**Published:** 2018-11-29

**Authors:** Filippo Biscarini, Fiorentina Palazzo, Federica Castellani, Giulia Masetti, Lisa Grotta, Angelo Cichelli, Giuseppe Martino

**Affiliations:** 1 Institute for Biology and Biotechnology in Agriculture (IBBA), CNR, Milano, Italy; 2 School of Medicine, Cardiff University, Cardiff, United Kingdom; 3 Faculty of Bioscience and Technology for Food, Agriculture and Environment, Università di Teramo, Teramo, Italy; 4 Bioinformatics Unit, PTP Science Park, Lodi, Italy; 5 Department of Medical and Oral Sciences and Biotechnologies, Università degli Studi “Gabriele d’Annunzio”, Chieti, Italy; University of Illinois, UNITED STATES

## Abstract

The rumen microbiome is fundamental for the productivity and health of dairy cattle and diet is known to influence the rumen microbiota composition. In this study, grape-pomace, a natural source of polyphenols, and copper sulfate were provided as feed supplementation in 15 Holstein-Friesian calves, including 5 controls. After 75 days of supplementation, genomic DNA was extracted from the rumen liquor and prepared for 16S rRNA-gene sequencing to characterize the composition of the rumen microbiota. From this, the rumen metagenome was predicted to obtain the associated gene functions and metabolic pathways in a cost-effective manner. Results showed that feed supplementations did alter the rumen microbiome of calves. Copper and grape-pomace increased the diversity of the rumen microbiota: the Shannon’s and Fisher’s alpha indices were significantly different across groups (p-values 0.045 and 0.039), and Bray-Curtis distances could separate grape-pomace calves from the other two groups. Differentially abundant taxa were identified: in particular, an uncultured *Bacteroidales* UCG-001 genus and OTUs from genus *Sarcina* were the most differentially abundant in pomace-supplemented calves compared to controls (p-values 0.003 and 0.0002, respectively). Enriched taxonomies such as *Ruminiclostridium* and *Eubacterium* sp., whose functions are related to degradation of the grape- pomace constituents (e.g. flavonoids or xyloglucan) have been described (p-values 0.027/0.028 and 0.040/0.022 in Pomace vs Copper and Controls, respectively). The most abundant predicted metagenomic genes belonged to the arginine and proline metabolism and the two- component (sensor/responder) regulatory system, which were increased in the supplemented groups. Interestingly, the lipopolysaccharide biosynthetic pathway was decreased in the two supplemented groups, possibly as a result of antimicrobial effects. Methanogenic taxa also responded to the feed supplementation, and methane metabolism in the rumen was the second most different pathway (up-regulated by feed supplementations) between experimental groups.

## Introduction

The rumen microbiota is a preeminent microbial community in the gastrointestinal tract of ruminants. This finely regulated ecosystem is what makes it possible for ruminants to digest fibrous plant material (inedible for other livestock), use it as source of energy and other metabolites, and transform it into high-quality food. In dairy cattle, the rumen microbiome plays a key role in milk production [1], well-being and health of the animals [2, 3]. The development of culture-independent high-throughput next-generation sequencing techniques provids a breakthrough in the characterization and analysis of microbiomes [4], with the rumen microbiome being no exception [5]. In particular, 16S rRNA gene sequencing [6] is a powerful technique to identify and quantify (in relative terms) the taxonomic composition of the rumen microbial population [7]. From metataxonomics results, the associated metagenome and related metabolic functions can be predicted, based on relative abundances and using a database of microbial genes functional annotations [8, 9]. The variability of the rumen microbiota across animals and over time has been investigated in a number of studies e.g. [10–13]. The diet is known to alter the composition of the rumen microbiota [14, 15]. Specific feed supplements have been the object of experimental trials on the rumen microbiome: these include canola [16], probiotic bacteria [17], organic acids [18, 19]. Mostly, feed supplementations had the objective of counteracting the effects of the high-energy diets typical of dairy cows on the rumen pH, the composition of the rumen microbiota, and the health of the animals. Grape-pomace is the solid residue from grape processing for wine production. It has high content of tannins and polyphenols e.g. [20, 21], which are known to exert an antioxidant activity and were previously shown to reduce rumen methane emissions in late-lactation dairy cows milk-fat yield [22]. Coppers is an essential trace element in the diet of livestock, and was shown to alter the gastrointestinal microbial composition of lactating cows [23]. It is therefore of interest to further investigate the role of these two feed supplements on the rumen microbiome composition and function.

In this study, we supplemented the daily ration of Holstein calves with either copper or grape-pomace. A metataxonomic approach was adopted, based on the sequencing of the 16S ribosomal RNA gene. The rumen microbiome of calves has been characterized, and differences arising as a consequence of dietary supplementations (copper, pomace) explored. Furthermore, from the quantification of taxonomic relative abundances, the rumen metagenome has been reconstructed, and its functional profile predicted. In this paper, we adhered to the terminology for microbiome research proposed by Marchesi and Ravel [24]: microbiota is the collection of microorganisms in the rumen, metataxonomics their characterization through 16S rRNA gene sequencing, and microbiome is the combination of the microbiota, their genes, functions and surrounding habitat. This is the first work to specifically look at the effect of copper and grape-pomace feed supplementation on the rumen microbiota composition and function, and one of the few reports so far on predictive microbiome profiling in cattle. Grape-pomace is a byproduct of wine processing, and its use to feed livestock illustrates a potential application of circular economy to the agri-food industry.

## Materials and methods

### Animals and experimental treatments

The research work presented here was carried out within the framework of the project VINCARN (“Miglioramento delle carni bovine, suine e avicole attraverso l’utilizzo di sottoprodotti della filiera enologica per fini mangimistici”) approved by “Direzione Politiche Agricole e di Sviluppo Rurale” (Directorate of Agriculture) of Regione Abruzzo on 13/08/2014 (determination DH26/40, n. Prot. RA 218995). This research used animals and data from commercial farms which were handled following the national legislation on animal welfare (DL n. 126, 07/07/2011, EC Directive 2008/119/EC), and then slaughtered complying with the EU Regulation 1099/2009 on the protection of animals at the time of killing.

Fifteen Holstein-Friesian male calves were used in this study. All calves came from the same dairy herd in central Italy (Casoli, CH, Abruzzo), and were included in the experiment at the same time, when they were approximately 7 months old. The average starting weight of the calves was 263 ± 21kg (259 ± 26, 268 ± 17 and 257 ± 21 in the control, grape-pomace and copper groups respectively). Before the supplementation experiment, all calves received a standard basal diet, which consisted of mainly alfalfa haylage plus a custom-formulated concentrate (detailed composition in S1 Table) that was offered to the animals *ad libitum*. From the beginning of the experiment through its completion (75 days), calves received a standard finishing diet (detailed composition in S2 Table) plus: i) nothing (control group); ii) a 10% DM (Dry Matter) red grape-pomace supplementation (pomace-group); iii) 3g/100 L of copper supplementation as cupric sulphate in drinking water (copper-group). Details from the feedstuff analysis of the custom-formulated concentrate and finishing diet are reported in S1 and S2 Tables. The three groups had equal size, consisting of five calves each. After 75 days of dietary supplementation, calves were slaughtered, at average age 259 ± 2 days (approximately 8.5 months) and average weight 345 ± 26 kg, 350 ± 22 kg and 332± 20 kg in the control, pomace and copper groups. The rumen liquor was sampled upon slaughtering in the premises of the abattoir. Following Niu et al. (2016), 500 mL of rumen samples (consisting in a mixture of liquid and solid fractions) from the dorsal, central and ventral region of the rumen of each animal were collected, pooled and filtered through four layers of cheesecloth, and then collected in 50 mL tubes and stored at -20 Celsius degrees until DNA extraction. Rumen liquor was sampled within 60-90 minutes from slaughtering. The handling of the animals was carried out following EU and national legislation on animal welfare (EU directive 2008/119/EC; DL n. 126, 07/07/2011; EU Regulation 1099/2009).

### DNA extraction and 16S rRNA-gene sequencing

Frozen rumen fluid samples were thawed at room temperature. Five ml of rumen fluid were centrifuged at 15,000 × g and the supernatant was removed. The DNA was extracted from pellets as previously described [25]. Briefly, bacterial cells were lysed by bead-beating in the presence of 4% (w/v) sodium dodecyl sulfate (SDS), 500 mm NaCl, and 50 mm EDTA. Impurities were removed by precipitation with ammonium acetate, and the nucleic acids were recovered by precipitation with isopropanol. Metagenomic DNA was then purified via sequential digestions with RNase and proteinase K, followed by the use of QIAamp DNA Stool MiniKit columns (Qiagen). The integrity and the concentration of gDNA were verified using a 2200 TapeStation Genomic Screen Tape device (Agilent, Santa Clara, CA, USA) and Qubit (Life Technologies). Libraries for metataxonomics were prepared according to the Illumina 16S-metagenomic library-prep-guide using v3 Reagents kit and the NexteraXT indices kit (Illumina, San Diego). Briefly, genomic DNA was normalized to 5 ng/μL, and 2.5 μL were used for library preparation using primers for the V3-V4 regions of the 16S rRNA-gene [6]. Libraries size and quality were evaluated with the Agilent TapeStation 2200 and quantified on Qubit (Life Technologies), and were diluted to 10 pm in hybridization buffer (HT1) for the cluster generation on the Miseq. In order to reduce unbalanced and biased base compositions, 10% of PhiX control library was spiked into the amplicon pool. Libraries were sequenced on the Miseq using a 2x300 paired-end sequencing module (Illumina, San Diego). Sequencing was carried out in the facilities of PTP Science Park (www.ptp.it).

### Bioinformatics processing

Demultiplexed paired-end reads from 16S rRNA-gene sequencing were first checked for quality using FastQC [26] for an initial assessment. Forward and reverse paired-end reads were joined into single reads using the C++ program SeqPrep [27]. After joining, reads were filtered for quality based on: i) maximum three consecutive low-quality base calls (Phred < 19) allowed; ii) fraction of consecutive high-quality base calls (Phred > 19) in a read over total read length ≥ 0.75; iii) no “N”-labeled bases (missing/uncalled) allowed. Reads that did not match all the above criteria were filtered out. All remaining reads were combined in a single FASTA file for the identification and quantification of OTUs (operational taxonomic units). Reads were aligned against the SILVA closed reference sequence collection release 123, with 97% cluster identity [28, 29], applying the Cd-hit clustering algorithm [30]. A pre-defined taxonomy map of reference sequences to taxonomies was then used for taxonomic identification along the main taxa ranks down to the genus level (domain, phylum, class, order, family, genus). By counting the abundance of each OTU, the OTU table was created and then grouped at each phylogenetic level. OTUs with total counts lower than 15 in fewer than 2 samples were filtered out. All of the above steps, except the FastQC reads quality check, were performed with the QIIME open-source bioinformatics pipeline for microbiome analysis [31]. The command lines and parameters used to process 16S rRNA-gene sequence data are detailed in S1 Appendix.

### Alpha and beta diversity indices

The rumen microbial diversity was assessed within- (alpha diversity) and across- (beta diversity) samples. All indices (alpha and beta diversity) were estimated from the complete OTU table (at the OTU level), filtered for OTUs with more than 15 total counts distributed in at least two samples. Besides the number of observed OTUs directly counted from the OTU table, within-sample microbial richness, diversity and evenness were estimated using the following indices: Chao1 and ACE (Abundance-based coverage Estimator) for richness, Shannon, Simpson and Fisher’s alpha for diversity [32–37], Simpson E and Pielou’s J (Shannon’s evenness) for evenness [38]. The across-sample rumen microbiota diversity was quantified by calculating Bray-Curtis dissimilarities [39]. Prior to the calculation of the Bray-Curtis dissimilarities, OTU counts were normalized for uneven sequencing depth by cumulative sum scaling (CSS, [40]. Among groups (copper, grape-pomace, control) and pairwise Bray-Curtis dissimilarities were evaluated non-parametrically using the permutational analysis of variance approach (999 permutations; [41]). Details on the calculation of the mentioned alpha- and beta-diversity indices are reported in S2 Appendix.

### Metagenome prediction and functional profiling

From the taxonomic composition of the rumen microbiota it is possible to predict its functional profile, using a database of precomputed reference genomic profiles. An approach based on nearest neighbor identification with a minimum sequence similarity was used to link 16S rRNA-gene sequences and functional annotations of prokaryotic genomes [8], as implemented in the Tax4Fun *R* package [42] coupled with the SILVA reference sequence collection. From the predicted metagenome gene ontologies and metabolic pathways were obtained based on the Kyoto Encylopedia of Genes and Genomes (KEGG) reference database of genome annotations [43].

### Software

Reads from 16S rRNA-gene sequencing were processed with the QIIME pipeline [31], used also to estimate most diversity indices. The ACE index and sample-base rarefaction were estimated using own *Python* (https://github.com/filippob/Rare-OTUs-ACE.git) and *R* (https://github.com/filippob/sampleBasedRarefaction) scripts. The prediction of the metagenome from metataxonomy and the functional profiling of the rumen microbiome were carried out using the Tax4Fun *R* package [42]. Plots were generated using the ggplot2 *R* package [44]. Additional data handling was performed with the R environment for statistical computing [45].

## Results

### Sequencing metrics, rarefaction and taxonomy description

**Fig 1:**
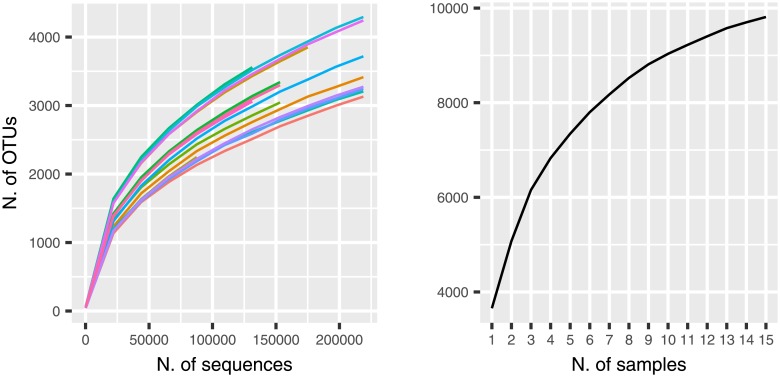
Rarefaction curves. Sequence-based (left) and sample-based (right) rarefaction curves for the sampled rumen microbiotas. Number of detected OTUs on the y-axis; number of sequences (left) and of samples (right) on the x-axis.

Sequencing the V3-V4 regions of the bacterial 16S rRNA gene produced a total of 8 393 698 reads (joined R1-R2 paired-end reads). After quality filtering, 2 772 892 sequences were removed, leaving 5 620 806 sequences for subsequent analyses (67% average retention rate, maximum 70%, minimum 60%). S3 Table reports reports the number of sequences before and after quality filtering using two quality thresholds: Phred > 3 (the default in the Qiime pipeline) and Phred > 19 (the threshold recommended by the Qiime manual and that was used in this work). A major difference in the number of sequences removed based on the quality score can be seen: 187 174 vs 2 772 892. However, the number of sequences retained after OTU picking (and successive filter on number of counts) is rather similar (4 141 362 vs 4 058 283): this indicates the robustness of the closed-reference OTU picking approach. On average, there were 475 652 (±236 180) sequences per sample in the control group, 339 125 (±169 147) in the copper-receiving group and 309383 (±159 021) in the pomace group. The initial number of OTUs identified was 13 257; after pruning out OTUs with less than 15 counts in at least 2 samples, 3 691 distinct OTUs were left. To check whether sequencing depth and sample size were adequate to characterize the composition of the rumen microbiota, sequence-based and sample-based rarefaction curves were generated from the OTU table before pruning (13257 OTUs). Sequence-based rarefaction curves were obtained from the QIIME pipeline [31]; the sample-based rarefaction curve was produced with ad hoc R functions (see: https://github.com/filippob/sampleBasedRarefaction). The observed number of OTUs detected was plotted as a function of the number of reads (up to 100 000) in each sample, and of the number of samples (Fig 1). Both curves tend to plateau asymptotically towards a maximum, indicating that sequencing depth and the number of samples were adequate to characterize the rumen microbiota in the present study. Deeper sequencing or the addition of any other samples would likely not increase significantly the number of new OTUs potentially discovered.

**Fig 2:**
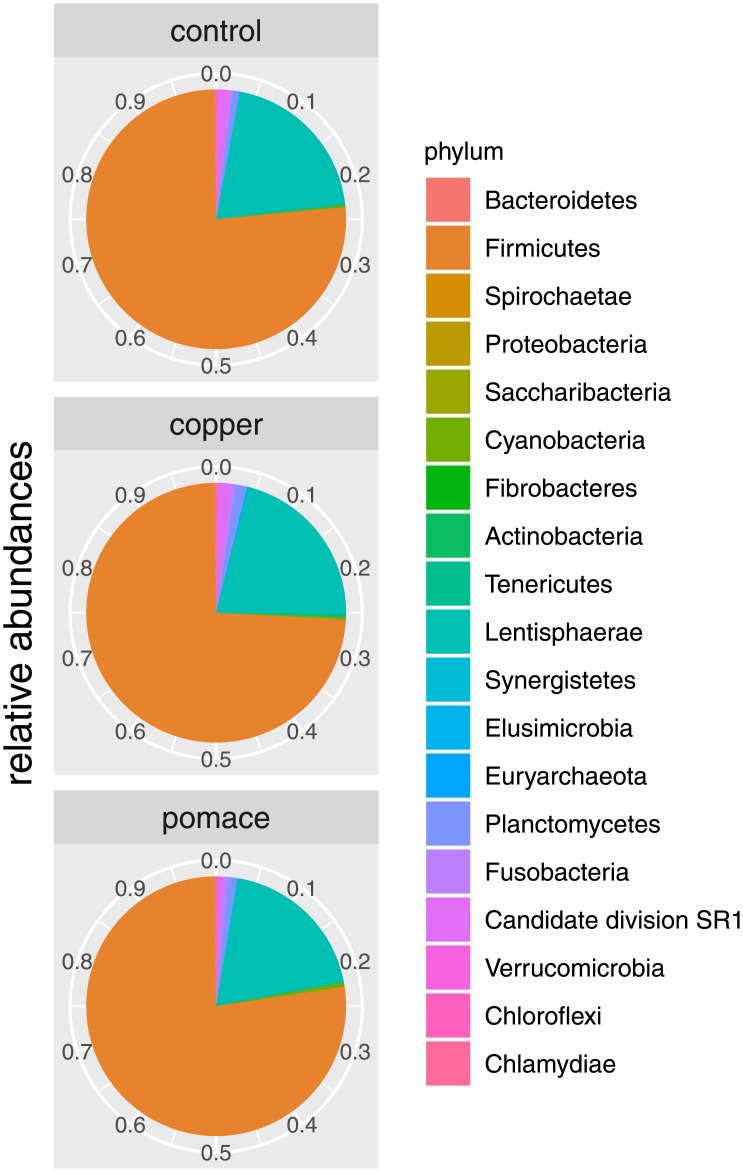
Relative abundances. Pie-chart of relative abundances for the phyla identified in the 15 calves rumen samples, grouped by dietary supplementation.

**Fig 3:**
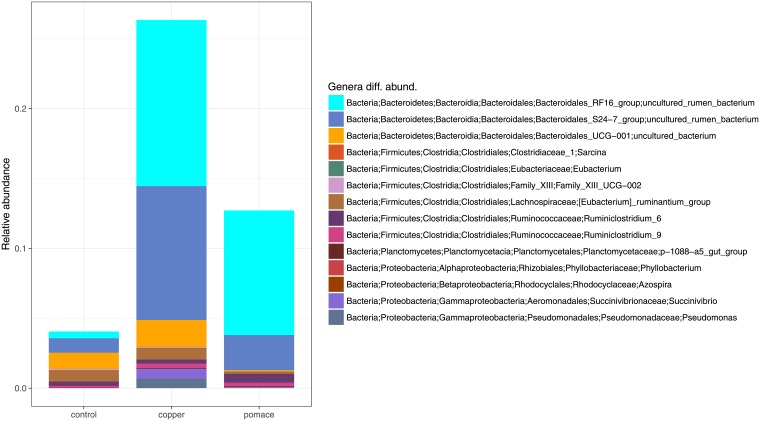
Bar-chart of the 14 genera with significant differential abundance in pairwise comparisons. Genera relative abundances in each group –control, copper, pomace– are reported, and the complete taxonomy of each genus is described.

OTUs were grouped taxonomically from the phylum to genus level (phylum, class, order, family, genus). The 3691 OTUs with more than 15 counts across samples clustered into 19 phyla, 39 classes, 60 orders, 94 families and 302 genera (see Table 1). The Bacteroidetes and Firmicutes phyla were the most abundant, representing respectively 75.7% (76.2%, 73.8%, 77.1%) and 20.0% (19.9%, 20.9%, 19.1%) of the total rumen microbiota (between brackets the proportions in the control, copper and grape-pomace groups). They were followed, at large distance, by *Spirochaetes* (1.27%: 1.50%/1.59%/0.73%), Proteobacteria (1.14%: 0.89%/1.39%/1.12%) and the phylum Saccharibacteria (0.42%: 0.23%/0.51%/0.52%). At deeper taxonomic levels, the rumen microbiota seemed to be dominated by few taxa: the orders Bacteroidales (75.3%: 75.9%/73.3%/76.7%) and Clostridiales (15.9%: 16.7%/15.9%/15.3%), the families Prevotellaceae (43.4%: 44.6%/39.8%/45.8%) and Rikenellaceae (14.4%: 9.45%/15.4%/18.4%), the genera Prevotella (44.6%: 44.6%/39.8%/45.8%) and Rikenellaceae RC9 gut group (13.7%: 8.90%/14.5%/17.6%). Fig 2 shows the pie chart of relative abundances of phyla in the three groups. Differences between groups in their rumen taxonomic composition (based on normalized counts) have been observed at the genus level (Table 2 with genera comparisons; Fig 3 with differences between groups in terms of relative abundances). Copper supplementation resulted in three differentially abundant taxa compared to controls, which comprised counts of the *Bacteroidales* S24-7 group (p-value = 0.03), *Planctomycetaceae* p-1088-a5 gut group (p-value = 0.022) and *Azospira* (p-value = 0.023). Out of ten genera differentially abundant, counts of *Bacteroidales* UCG-001 uncultured bacterium (p-value = 0.003) and of *Sarcina* (p-value = 0.000) were the most differentially abundant in the grape-pomace supplemented rumen compared to controls. Both supplementations shown a difference in the abundance of the *Planctomycetaceae* p-1088-a5 gut group counts compared to controls (p-value = 0.022 and p-value = 0.039, respectively). Four genera, all belonging to the phylum Firmicutes, were differentially abundant between grape-pomace and copper supplementations.

**Table 1:** Summary of identified taxonomies and estimated alpha diversity indices in the rumen microbiota of dairy calves from three experimental groups. P-values for among-group differences from analysis of variance.

taxa	control	copper	pomace	total	p-value
phylum	13	13	11	19	0.898
class	23	22	21	39	0.947
order	26	28	25	60	0.991
family	42	46	44	94	0.939
genus	130	142	129	302	0.905
observed_otus	2341.80	2369.40	2481.40		0.699
chao1	2648.84	2744.75	2829.40		0.409
ACE	2661.13	2739.26	2850.21		0.407
simpson	0.92	0.95	0.97		0.097
shannon	6.09	6.67	7.07		0.045
fisher_alpha	347.62	370.88	397.50		0.039
equitability	0.55	0.60	0.63		0.083
simpson_e	0.01	0.01	0.01		0.209

Results based on the 3691 distinct OTUs retrieved from the SILVA reference database release 123. ACE: Abundance-based Coverage Estimator; Equitability: Shannon evenness; Simpson_e: Simpson evenness. *: p-values < 0.1 and **: p-values < 0.05.

**Table 2:** Comparison of CSS-normalized OTU counts among groups, at the genus taxonomic level.

Comparison	Taxonomy	p-value	SEM
Control vs. Copper	Bacteroidales S24-7 group;uncultured rumen bacterium	0.033	0.858
	Planctomycetaceae; p-1088-a5 gut group	0.022	0.005
	Azospira	0.023	0.000
Control vs. Pomace	Bacteroidales RF16 group;uncultured rumen bacterium	0.040	0.800
	Bacteroidales UCG-001;uncultured bacterium	0.003	0.034
	Sarcina	0.000	0.001
	Eubacterium	0.022	0.001
	Ruminiclostridium 6	0.040	0.060
	Ruminiclostridium 9	0.040	0.022
	Planctomycetaceae;p-1088-a5 gut group	0.039	0.005
	Phyllobacterium	0.019	0.001
	Succinivibrio	0.011	0.004
	Pseudomonas	0.019	0.003
Pomace vs. Copper	Eubacterium	0.028	0.001
	Clostridiales; Family XIII UCG-002	0.016	0.005
	Lachnospiraceae;[Eubacterium] ruminantium group	0.015	0.076
	Ruminiclostridium 6	0.027	0.032

CSS: cumulative sum-scaling; SEM: standard error of the mean $\left(\sqrt{\frac{\sigma_{1}^{2}}{n_{1}}+\frac{\sigma_{2}^{2}}{n_{2}}}\right)$

### Diversity indices

**Fig 4:**
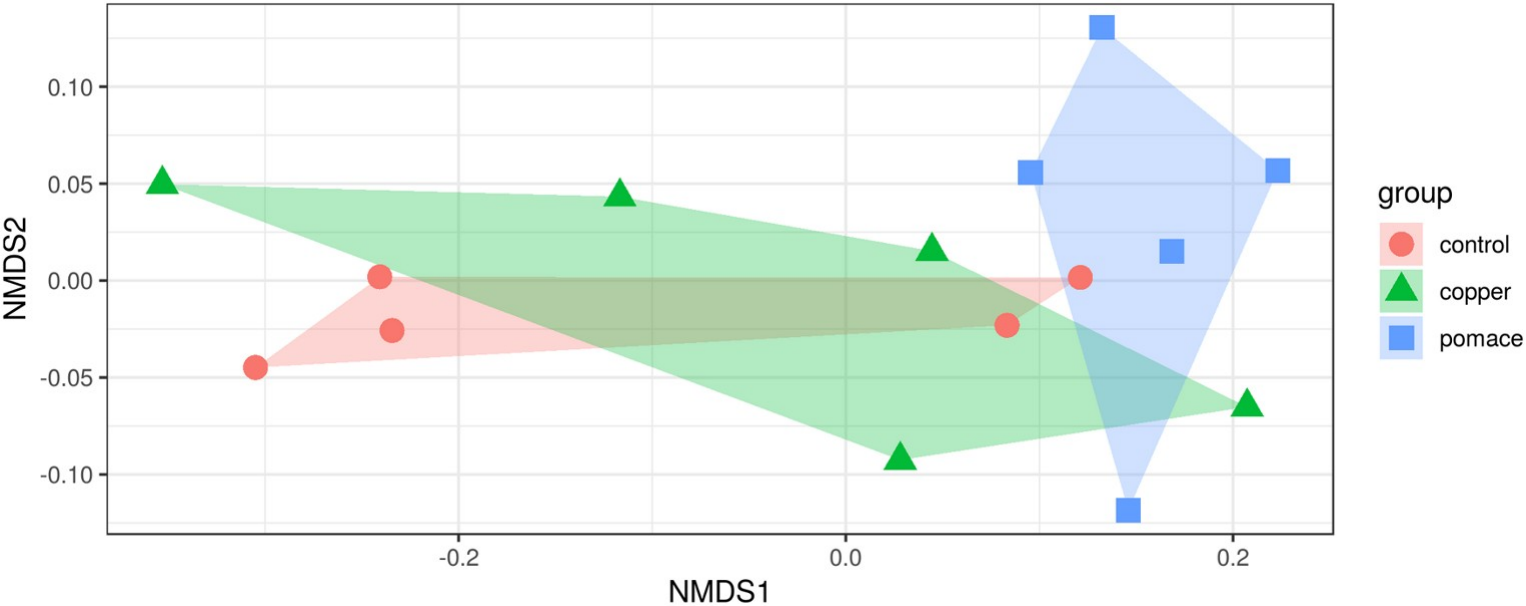
First two dimensions from the (non-metric) multi-dimensional scaling of the Bray-Curtis dissimilarity matrix. Samples were grouped by experimental unit. PERMANOVA amongst all groups p = 0.053 (using 999 permutations); pairwise PERMANOVA: copper-pomace p = 0.035; copper-control and pomace-control p-value > 0.05.

The estimated alpha diversity indices for describing the richness, diversity and evenness of the rumen microbiota in the three experimental groups are reported in Table 1. The richness estimators Chao1 and ACE did not show differences among groups, as well as the average number of observed OTUs, and the evenness estimator Simpson_e. A mildly significant difference was observed in the equitability index as a measure of evenness of the microbial communities (p-value < 0.10). On the contrary, the diversity indices Shannon (p-value < 0.05) and Fisher’s alpha (*p* − *value* < 0.05) showed a significant difference among groups. The rumen microbiota of grape pomace-fed calves had higher diversity compared to copper-fed calves (intermediate alpha diversity) and controls. The Bray-Curtis dissimilarity index was estimated from OTU counts to measure diversity across samples (beta diversity). The first two dimensions from the non-metric multidimensional scaling of the Bray-Curtis dissimilarity matrix (Fig 4) reveals a slightly significant different distance between the three groups (p-value = 0.053, from 999 permutations of the analysis of variance). In particular, a significant difference of the ruminal bacterial communities organization has been observed between supplementations (p-value = 0.035), but not between each supplementations and control (p-value > 0.05).

### Predictive functional profiling of the rumen microbiota

**Fig 5:**
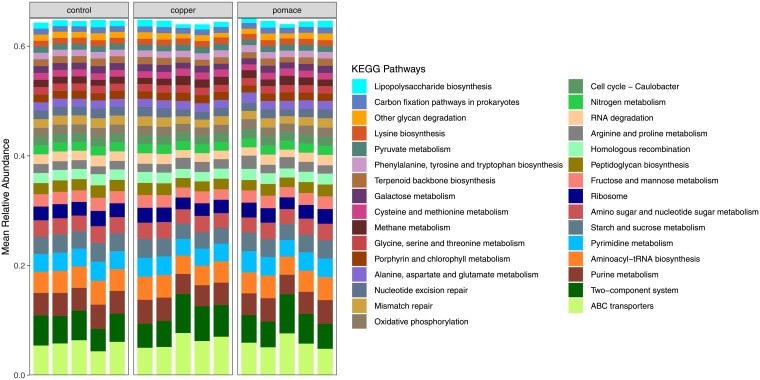
KEGG metabolic pathways identified from the predicted metagenome for each sample (calf). Only pathways with average relative abundance > 1% across samples were included.

**Fig 6:**
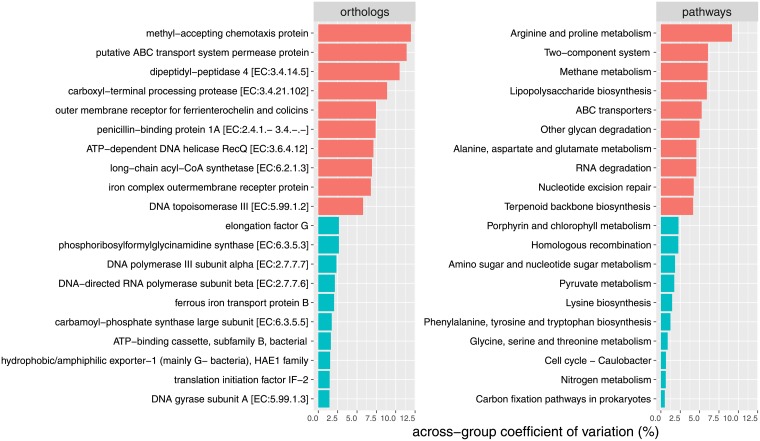
Differential ortholog genes and metabolic pathways. Most (top, red) and least (bottom, blue) different (measured as across-group coefficient of variation) ortholog genes (left) and metabolic pathways (right) among the three experimental groups.

From the predicted metagenome, 6449 ortholog genes, involved in 280 metabolic pathways were retrieved. The most abundant genes were the iron-complex outer-membrane receptor protein, the bacterial ATP-binding cassette (ABC transporter), and the hydrophobic/amphiphilic exporter-1 (Table 3). Fig 5 reports the most represented (average relative abundance >1% across samples) metabolic pathways for each calf; pathways are ordered by decreasing relative abundance (from bottom to top). The top three pathways include ABC transporters (across-membrane cellular transportation of substrates), the two-component (sensor/responder) regulatory system, and purine metabolism. For each metabolic pathway and ortholog gene, the coefficient of variation of relative abundance across groups (control, copper, pomace) was calculated. The metabolic pathways and genes with the largest (top 10) and smallest (bottom 10) variation among groups are listed in Fig 6: the most variable genes and pathways include, respectively: the methyl-accepting chemotaxis protein, the putative ABC transport system permease protein and the dipeptidyl-peptidase 4; arginine and proline metabolism, the two-component (sensor/responder) regulatory system, and methane metabolism.

**Table 3:** Top 10 (most abundant) genes and pathways from metagenome prediction in the 15 calves samples.

KeggID	gene name	KeggID	pathway
K02014	iron complex outermembrane recepter protein	KO02010	ABC transporters
K06147	ATP-binding cassette, subfamily B, bacterial	KO02020	Two-component system
K03296	hydrophobic/amphiphilic exporter-1 (mainly G- bacteria), HAE1 family	KO00230	Purine metabolism
K05349	beta-glucosidase [EC:3.2.1.21]	KO00970	Aminoacyl-tRNA biosynthesis
K01190	beta-galactosidase [EC:3.2.1.23]	KO00240	Pyrimidine metabolism
K03701	excinuclease ABC subunit A	KO00500	Starch and sucrose metabolism
K02004	putative ABC transport system permease protein	KO00520	Amino sugar and nucleotide sugar metabolism
K03406	methyl-accepting chemotaxis protein	KO03010	Ribosome
K03088	RNA polymerase sigma-70 factor, ECF subfamily	KO00051	Fructose and mannose metabolism
K03737	putative pyruvate-flavodoxin oxidoreductase [EC:1.2.7.-]	KO00550	Peptidoglycan biosynthesis

## Discussion

The characterization of the rumen microbiota and its functional profile from 15 calves fed different dietary supplementations has been presented here. The sequencing of the V3-V4 variable regions of the rRNA gene (16S subunit) appeared to be adequate as shown by the asymptotic plateauing of both sequence- and sample-based rarefaction curves (Fig 1). The sensitivity analysis of sequence quality filtering (comparisons of Phred quality score thresholds: Phred > 3 vs Phred > 19) indicated an overall robustness of results from the closed-reference OTU picking approach; however, a stricter quality filtering (Phred > 19) is likely to remove most poor quality sequences and, consequently, most spurious OTUs.

Overall, the prevailing bacterial phyla were Bacteroidetes and Firmicutes, distantly followed by Spirochaetes and Proteobacteria: this is a common finding from the cattle rumen microbiota as reviewed by Morgavi et al. [2] and reported in more recent research results [19, 46]; it is also in line with the age development of rumen microbial communities in calves, which feature decreasing Proteobacteria and simultaneously increasing Bacteroidetes and Firmicutes from birth to weaning [11]. The most abundant genera, *Prevotella* spp and *Rikenellaceae RC9 gut group* spp varied substantially across samples (coefficient of variation 40.5% and 96.6% respectively), though no significant difference between groups were observed (p-value > 0.60 in both cases).

### Effect of feed supplementation

The main objective of the experiment was to investigate differences in the rumen microbiota arising as a consequence of different dietary supplementations: the addition of either the mineral micro-element copper or grape-pomace to the feed ration was compared to unsupplemented control animals. Previous works have looked at the effect of different feed supplementations on the rumen microbiome in dairy cattle. Golder et al. [16] found that the addition of canola meal to the ration clearly differentiated the rumen microbiota from that of control animals. De Nardi et al. [19] found higher microbial richness and diversity (Fisher’s alpha index) when supplementing the ration with dicarboxylic acids or polyphenols. In goats, rhubarb (*Rheum officinale*) root meal supplementation was reported to increase the richness of the rumen microbiota (Chao1 index, [47]). Here, we found that microbial richness in the rumen was barely affected by supplementations (slightly more richness with grape-pomace, no effect of copper); on the other hand, microbial diversity (Shannon and Fisher’s alpha indices) clearly increased with copper and, mainly, grape-pomace supplementation. Between-sample distances based on the rumen microbiota composition revealed that the grape-pomace group appeared to be relatively clearly separated from the control and copper groups, which conversely overlapped substantially. Moate and collaborators reported a rumen microbiota shift in dried grape marc or ensiled grape marc supplemented cows compared to control diet, with no differences reported between the type of supplementation [22]. However, they did not report taxonomic differences from such supplementations trial, as they used the terminal restriction fragment (T-RF) length polymorphism for characterizing bacterial and archaeal (amongst other) community structures [48].

When looking at specific taxa (Table 1), unclassified genera from the *Bacteroidales S24-7* and *RF16* groups were enriched in the microbiota of copper and grape-pomace supplemented calves, respectively. De Nardi et al. [19] found an enrichment of the order Bacteroidales in the rumen of polyphenol-supplemented dairy heifers. Recently, Popova and colleagues [49] observed a reduction in unclassified *Bacteroidales S24-7* genera in the rumen of young Charolais bulls receiving a linseed plus nitrate supplementation. OTUs from genus *Ruminiclostridium 6* and *Eubacterium* were more abundant in the rumen of grape-pomace supplemented calves compared to copper supplementation and controls. *Ruminiclostridium cellulyticum* has the ability to degrade branched plant polysaccharide such as xyloglucan [50], which actually is present in the grape-pomace cell wall [51]. Resulting oligosaccharides were shown to be imported in the cytoplasm through the ATP-binding cassette (ABC) transporter, to be further sequentially degraded into a final product of glucose and glucose-1-phosphate [50]. *Eubacterium* sp. Were shown to degrade flavonoids in human and rat feces [52]. Kasparkova et al. [53] reported a negative correlation between ruminal Eubacteriaceae counts and levels of the isoflavone-extract daidzein, following the administration of isoflavone-rich feed in lactating cows.

The Firmicutes:Bacteroidetes ratio in the gut microbiota is known to play a role in adipogenesis: Jami et al [1] observed a strong positive correlation between this ratio and milk-fat yield. In studies on obesity in mice and humans, it has been related to higher blood and tissue fat [54, 55]. The role of adipogenesis in the autoimmune Graves’ orbitopathy has been established [56], and the relationship between the gut microbiome and fat metabolism in this disease is object of current research (EU “Indigo” project: www.indigo-iapp.eu/). In the present study, the Firmicutes to Bacteroidetes ratio was 0.28 in controls, 0.31 in copper-supplemented calves, and 0.25 in grape-pomace-supplemented calves. The difference is small, but appears to point to a possible reduction in the ratio between Firmicutes and Bacteroidetes induced by the supplementation with grape-pomace.

**Fig 7:**
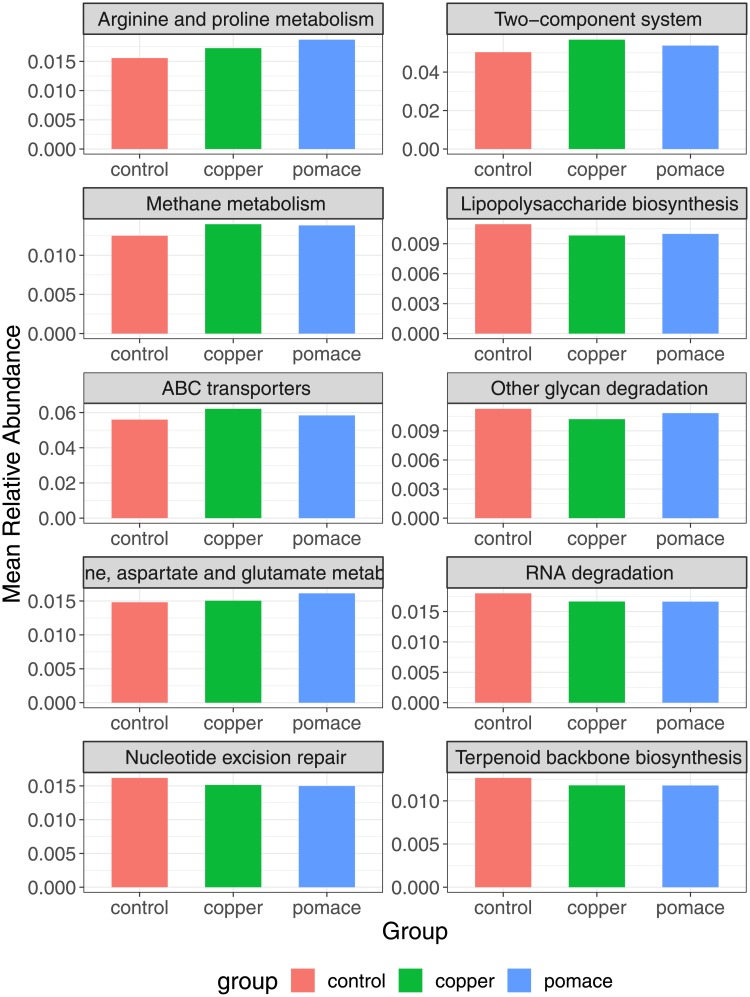
Most variable metabolic pathways among experimental groups. Relative abundances on the y-axis.

From the metagenome prediction, the majority of genes belonged to membrane transport, carbohydrate metabolism and replication and repair functions. Similar results were obtained from a previous report on the functional analysis of the rumen microbiota in dairy cattle [46]. Across experimental groups, the most differentially abundant genes and pathways -with a coefficient of variation ≥ 9% were: the methyl-accepting chemotaxis protein involved in bacterial motility; the enzyme dipeptidyl-peptidase 4 involved in protein digestion and absorption; the ABC membrane transport system permease protein, member of a superfamily of transmembrane proteins present in all extant phyla, from prokaryotes to mammals; and the arginine and proline metabolism (higher in the grape-pomace group, followed by the copper and control groups, see Fig 7). The two-component system -a signal transduction systems that enables bacteria to sense, respond, and adapt to changes in their environment- was more abundant in the copper-supplemented group. Interestingly, the long-chain acyl-CoA synthetase gene, which plays a role in the lipid metabolism, and the lipopolysaccharide biosynthesis metabolic pathway were found to be under-represented in the copper and grape-pomace groups: this finding is consistent with the lower Firmicutes:Bacteroidetes ratio, and may contribute to explain why the latter is associated with the down-regulation of the fat metabolism. Additionally, a reduction in the lipopolysaccharide (LPS) biosynthetic pathway may be related to a reduced growth of possible pathogenic bacteria for humans (e.g. *Salmonella*, *Escherichia coli*), as a consequence of the antimicrobial activity of the supplementations, as suggested also by De-Nardi [19]. A reduction in Enterobacteriaceae (including *Salmonella*, *Shigella*, *E*.*coli*) after grape-pomace supplementation has been described in lamb fecal samples [57].

Overall, grape-pomace supplementation appeared to induce a larger effect than copper supplementation on the rumen microbiome, in terms of both alpha- and beta-diversity indices, number of differentially abundant taxa, and functional profile.

### Methods for functional profiling of the microbiome

The possibility of predicting the metagenome and associated functional annotations from 16S rRNA gene sequencing data is a major breakthrough in the analysis of microbial communities. Deep shotgun metagenomics sequencing is the current golden standard to precisely characterize communities’ metagenomes [58], especially to detect rare organisms and genes; however, this approach is often prohibitively expensive, and sometimes challenging to analyse: therefore, metagenome prediction offer a valid cost-effective alternative for the functional profiling of microbiotas. Aßhauer and Meinicke [8] proposed a method to predict functional profiles from 16S rRNA data, and implemented it into the R package “Tax4Fun” [42]. This method and its implementation are based on the SILVA rRNA reference database [28]. In parallel, an alternative method -named PICRUSt (Phylogenetic Investigation of Communities by Reconstruction of Unobserved States)- for the predictive functional profiling of microbial communities from 16S rRNA marker gene data has been developed and implemented in a Python package [9]. PICRUSt is based on the Greengenes 16S rRNA sequence database [59].

Such a technique has been recently applied also in the description of the functional role of the rumen and cecal microbiota of Charolais bulls, which were found to be highly similar [49]. In this work, the approach based on Tax4Fun and SILVA was used to predict the metagenome from metataxonomy, and results have been presented. Alongside, data were re-analysed also using PICRUSt plus the Greengenes database, and results from the two pipelines were compared. From the Tax4Fun-SILVA pipeline 6449 unique KEGG items (ortholog genes and pathways) were retrieved; from PiCrust-Greengenes the number of retrieved unique items was 6909. Across the two sets of results, 5983 items were in common (92.8% relative to the Tax4Fun-SILVA pipeline), indicating that the two approaches yielded very similar results. This gives robustness to the presented functional profiles of the rumen microbiota in calves, and contributes to increase confidence in the gene functional analysis based on metagenome prediction.

### Methanogenic taxa

Dietary treatments are known to exert an effect on rumen methane production and to alter the composition of the rumen microbiota (see Negussie et al. [60] for a review—S1 Table). Many methanogens are known to belong to the Archaea domain [61]. We found a small proportion of Archaeal taxonomies described from the V3-V4 primers, and calculated the Pearson correlation between rumen bacteria and Archaea counts in the three experimental groups. This correlation was positive in the control (0.648) and copper (0.672) groups (the more the bacteria, the more the archaea), but negative in the grape-pomace group (-0.231: the larger the bacteria counts, the fewer the archaea). Specific methanogenic taxa (taken from a review by Tapio et al. [62]) like those belonging to the phylum Euryarchaeota, which included methanogens genera such as *Methanomicrococcus* and *Methanosphaera*, have been identified and quantified (Table 4). Results were somewhat ambiguous: although the minimum count was mostly observed in the grape-pomace (11 out of 18 times) and, secondarily, the copper (9 out of 18 times) groups, sometimes the control group had the minimum methanogens counts (3 out 18 times). It should be emphasized, though, that Archaea were scarcely represented in the sequenced reads, accounting for only 64 out of the 13258 detected OTUs, and we resorted to the unfiltered OTU table to present these results; this was probably due to the choice of the V3-V4 primers used, not specifically designed to target Archaea. In no case was the difference among groups statistically significant. Methane metabolism was the second most different metabolic pathway across experimental groups (coefficient of variation ∼ 6%): this pathway was overrepresented in the grape-pomace and copper groups, relative to controls. Summarizing, feed supplementations, especially grape-pomace, appear to have an influence on methanogenic rumen microbial taxa (in line with findings by Moate et al [22]); however, it is not clear in which direction, since mixed results were obtained from different analyses (Archaea-bacteria correlations, specific methanogenic taxa, pathway analysis).

**Table 4:** Counts of methanogenic taxa in the gut microbiota: Comparison between experimental groups.

domain	phylum	class	order	family	genus	species	control	rame	vinacce	tot	p-value
Archaea	Euryarchaeota	Methanobacteria	Methanobacteriales	Methanobacteriaceae	Methanobrevibacter	uncultured archaeon	183	53	263	499	0.00
Archaea	Euryarchaeota	Methanobacteria	Methanobacteriales	Methanobacteriaceae	Methanobrevibacter	uncultured Methanobrevibacter sp.	73	10	36	119	0.27
Archaea	Euryarchaeota	Methanobacteria	Methanobacteriales	Methanobacteriaceae	Methanobrevibacter	uncultured methanogenic archaeon	1	3	4	8	0.36
Archaea	Euryarchaeota	Methanobacteria	Methanobacteriales	Methanobacteriaceae	Methanobrevibacter	uncultured rumen methanogen	35	58	9	102	0.15
Archaea	Euryarchaeota	Methanobacteria	Methanobacteriales	Methanobacteriaceae	Methanosphaera	uncultured archaeon	8	5	0	13	0.44
Archaea	Euryarchaeota	Methanomicrobia	Methanosarcinales	Methanosaetaceae	Methanosaeta	uncultured archaeon	2	1	0	3	0.36
Archaea	Euryarchaeota	Methanomicrobia	Methanosarcinales	Methanosarcinaceae	Methanimicrococcus	unidentified methanogen ARC45	0	2	0	2	0.40
Archaea	Euryarchaeota	Thermoplasmata	Thermoplasmatales	Thermoplasmatales Incertae Sedis	Candidatus Methanomethylophilus	Methanoculleus sp. CAG:1088	2	0	0	2	0.40
Archaea	Euryarchaeota	Thermoplasmata	Thermoplasmatales	Thermoplasmatales Incertae Sedis	Candidatus Methanomethylophilus	uncultured archaeon	4	0	1	5	0.14
Archaea	Euryarchaeota	Thermoplasmata	Thermoplasmatales	Thermoplasmatales Incertae Sedis	Candidatus Methanomethylophilus	uncultured methanogenic archaeon	2	0	2	4	0.62
Bacteria	Bacteroidetes	Bacteroidia	Bacteroidales	Prevotellaceae	Prevotella 1	Prevotella ruminicola	275	15	161	451	0.35
Bacteria	Firmicutes	Clostridia	Clostridiales	Ruminococcaceae	Faecalibacterium	bacterium ic1379	1	0	1	2	0.62
Bacteria	Firmicutes	Clostridia	Clostridiales	Ruminococcaceae	Faecalibacterium	Faecalibacterium cf. prausnitzii KLE1255	1	0	1	2	0.62
Bacteria	Firmicutes	Clostridia	Clostridiales	Ruminococcaceae	Faecalibacterium	Faecalibacterium prausnitzii	1	1	0	2	0.62
Bacteria	Firmicutes	Clostridia	Clostridiales	Ruminococcaceae	Faecalibacterium	human gut metagenome	0	1	0	1	0.40
Bacteria	Firmicutes	Clostridia	Clostridiales	Ruminococcaceae	Faecalibacterium	Trichuris trichiura (human whipworm)	11	17	7	35	0.24
Bacteria	Firmicutes	Clostridia	Clostridiales	Ruminococcaceae	Faecalibacterium	uncultured bacterium	4933	4242	4018	13193	0.58
Bacteria	Firmicutes	Clostridia	Clostridiales	Ruminococcaceae	Faecalibacterium	uncultured bacterium adhufec365	1	0	0	1	0.40
Bacteria	Firmicutes	Clostridia	Clostridiales	Ruminococcaceae	Faecalibacterium	uncultured Faecalibacterium sp.	5	7	1	13	0.15
Bacteria	Firmicutes	Clostridia	Clostridiales	Ruminococcaceae	Faecalibacterium	uncultured low G+C Gram-positive bacterium	0	0	1	1	0.40
Bacteria	Firmicutes	Clostridia	Clostridiales	Ruminococcaceae	Faecalibacterium	uncultured organism	116	83	96	295	0.45
Bacteria	Firmicutes	Clostridia	Clostridiales	Ruminococcaceae	Faecalibacterium	uncultured rumen bacterium	4	13	1	18	0.53

Methanogenic taxa were obtained from a review by Tapio et al. [62]; counts were 810 obtained from the unfiltered OTU table.

## Conclusions

The sequencing of the 16S rRNA marker gene constitutes an extraordinary advancement in the genetic analysis of microbial communities. Coupling metataxonomics with metagenome prediction gives insights into the genes and metabolic pathways associated with a microbiome, and is a very powerful technique for the functional profiling of microbial communities. Here, it was applied to the profiling of the rumen microbiome in dairy calves fed differentially supplemented diets. Copper and grape-pomace feed supplementations appeared to alter the rumen microbiome, both in terms of species diversity and gene functions. Results were in line with previous findings in both ruminal, human and murine microbiota. The addition of grape-pomace, in particular, seemed to modify the rumen microbial population, with an apparent effect also on methanogenic bacteria and methane metabolism in the rumen. It needs be emphasized though, that Archaea and methanogenic taxa were not specifically targeted by the sequencing approach employed in this study, and results should therefore be considered as indicative.

Overall, although from a small-scale experiment, the results presented here offer an interesting characterization of the rumen microbiota in dairy calves and the effects that copper and grape-pomace feed supplementation may exert. Grape-pomace, in particular, is a common byproduct from wine processing, and knowledge of its effects on the rumen microbiome will be helpful in assessing its potential as feedstuff for livestock. Using local industrial byproducts as animal feed constitutes a nice example of circular economy applied to the agri-food industry. Further experiments are however needed to confirm the neutral-to-positive effects of grape-pomace on the bovine rumen microbiome.

## Supporting information

S1 Table Custom-formulated concentrate. Ingredients and nutrient composition of the custom-formulated concentrate, which was fed to all calves prior to the feed supplementation experiment.
(PDF)

S2 Table Finishing diets. Ingredients and nutrient composition of the finishing diets fed to the three experimental groups (control, grape pomace and copper supplementation).
(PDF)

S3 Table Sequencing metrics. Number of 16S rRNA gene sequences retained after successive steps of the bioinformatics processing and filtering: comparison between two quality filtering thresholds (Phred>3, default in the Qiime pipeline; Phred>19, threshold used in this study).
(PDF)

S1 Appendix Metataxonomics pipeline command lines. Specific command lines used in the Qiime bioinformatics pipeline to process 16S rRNA-gene sequencing data.
(PDF)

S2 Appendix Alpha- and beta-diversity indices. Break out of the calculations involved in the estimation of the alpha- and beta-diversity indices used in this study.
(PDF)
