# Supplementary material for: Rumen microbiome in dairy calves fed copper and grape-pomace dietary supplementations: Composition and predicted functional profile
Source: PLoS One. 2018 Nov 29;13(11):e0205670. doi: 10.1371/journal.pone.0205670 (PMC6264861; doi:10.1371/journal.pone.0205670)
Supplement: S1 Table — Ingredients and nutrient composition of the custom-formulated concentrate, which was fed to all calves prior to the feed supplementation experiment. (PDF) [file pone.0205670.s001.pdf]

Functional profiling of the rumen microbiota in dairy calves fed copper and grape-pomace dietary supplementations

Filippo Biscarini[1,2] et al.*,

1 Institute for Biology and Biotechnology in Agriculture (IBBA), CNR, Milano, Italy
2 School of Medicine, Cardiff University, Cardiff, United Kingdom
*E-mail: gmartino@unite.it

Ingredient	%
Wheat meal	41.99
Corn meal	9.00
Barley	9.00
Chopped wheat	8.00
Sunflower meal 30%	7.80
Wheat bran	5.00
Scraps cookies	4.00
Corn	3.66
Sunflower meal 36%	3.00
Betamel	1.50
Soybean meal 44%	1.50
Sugarcane molasses	1.50
Soybean meal 47%	0.84
$CaCO_3$	3.00
NaCl 39%	0.80
Vitamins and minerals mix	0.40

Nutrient composition, % DM basis	
DM	88.05
CP	14.16
Fat	3.32
Fiber	7.54
NDF	18.49
Ash	6.64
Ca	1.00
P	0.42
NE (Mcal/kg)	0.89

S1 Table: Ingredients and nutrient composition of the custom-formulated concentrate, which was fed to all calves prior to the feed supplementation experiment. DM: dry matter; CP: crude protein; NDF: neutral-detergent fiber; NE: net energy
