# Supplementary material for: Rumen microbiome in dairy calves fed copper and grape-pomace dietary supplementations: Composition and predicted functional profile
Source: PLoS One. 2018 Nov 29;13(11):e0205670. doi: 10.1371/journal.pone.0205670 (PMC6264861; doi:10.1371/journal.pone.0205670)
Supplement: S2 Table — Ingredients and nutrient composition of the finishing diets fed to the three experimental groups (control, grape pomace and copper supplementation). (PDF) [file pone.0205670.s002.pdf]

Functional profiling of the rumen microbiota in dairy calves fed copper and grape-pomace dietary supplementations

Filippo Biscarini[1,2] et al.*,

1 Institute for Biology and Biotechnology in Agriculture (IBBA), CNR, Milano, Italy
2 School of Medicine, Cardiff University, Cardiff, United Kingdom
*E-mail: gmartino@unite.it

Ingredient %	Control	Grape.pomace	Copper
Wheat meal	14	19.99	14
Corn meal	12	19.3	12
Corn	10	10	10
Chopped wheat	10	8	10
Sunflower meal	10	4.1	10
Bean	7.5	8	7.5
Scraps cookies	5.35	5	5.35
Barley meal	5	-	5
Wheat bran	5	5	5
Corn distillers grain	5	1	5
Barley	3.7	2	3.7
Citrus pulp	3.5	-	3.5
Soybean meal	2.95	1.4	2.95
Sugarcane molasses	1	1	1
Soybean hulls	0.6	1	0.6
Grape pomace	-	10	-
$CaCO_3$	2.5	1.65	2.5
NaCl 39%	0.6	0.6	0.6
NaHCO3 27%	0.5	0.8	0.5
Soybean oil	0.36	-	0.36
CaHPO4 17%	-	0.61	-
Vitamins and minerals mix	0.44	0.55	0.44
Nutrient composition, % DM basis			
DM	88.59	88.59	88.59
CP	15.99	15.81	15.99
Fat	3.75	4.52	3.75
Fiber	8.51	8.18	8.51
NDF	20.87	20.23	20.87
Ash	7.5	8.5	7.5
Ca	1.13	1.13	1.13
P	0.47	0.51	0.47
NE (Mcal/kg)	0.97	0.99	0.97

S2 Table: Ingredients and nutrient composition of the finishing diets fed to the three experimental groups (control, grape pomace and copper supplementation).
