# Supplementary material for: Rumen microbiome in dairy calves fed copper and grape-pomace dietary supplementations: Composition and predicted functional profile
Source: PLoS One. 2018 Nov 29;13(11):e0205670. doi: 10.1371/journal.pone.0205670 (PMC6264861; doi:10.1371/journal.pone.0205670)
Supplement: S3 Table — Number of 16S rRNA gene sequences retained after successive steps of the bioinformatics processing and filtering: comparison between two quality filtering thresholds (Phred>3, default in the Qiime pipeline; Phred>19, threshold used in this study). (PDF) [file pone.0205670.s003.pdf]

Functional profiling of the rumen microbiota in dairy calves fed copper and grape-pomace dietary supplementations

Filippo Biscarini[1,2] et al.*,

1 Institute for Biology and Biotechnology in Agriculture (IBBA), CNR, Milano, Italy
2 School of Medicine, Cardiff University, Cardiff, United Kingdom
*E-mail: gmartino@unite.it

step	Phred3	Phred19
multiple_extract_barcodes	16787720	16787720
multiple_join_paired_ends	8393698	8393698
multiple_split_library	8206524	5620806
closed_otupicking	4164028	4089545
filter_otus	4141362	4058283

S3 Table: Number of 16S rRNA gene sequences retained after successive steps of the bioinformatics processing and filtering: comparison between two quality filtering thresholds (Phred¿3, default in the Qiime pipeline; Phred¿19, threshold used in this study).
