# Supplementary material for: Rumen microbiome in dairy calves fed copper and grape-pomace dietary supplementations: Composition and predicted functional profile
Source: PLoS One. 2018 Nov 29;13(11):e0205670. doi: 10.1371/journal.pone.0205670 (PMC6264861; doi:10.1371/journal.pone.0205670)
Supplement: S2 Appendix — Break out of the calculations involved in the estimation of the alpha- and beta-diversity indices used in this study. (PDF) [file pone.0205670.s005.pdf]

Functional profiling of the rumen microbiota in dairy calves fed copper and grape-pomace dietary supplementations

Filippo Biscarini[1,2] et al.*,

1 Institute for Biology and Biotechnology in Agriculture (IBBA), CNR, Milano, Italy
2 School of Medicine, Cardiff University, Cardiff, United Kingdom
*E-mail: gmartino@unite.it

APPENDIX 2: ALPHA-AND BETA-DIVERSITY INDEXES

Alpha diversity

Richness indexes : The Chao1 and Abundance-based Coverage Estimator (ACE) richness indexes were calculated as described by Chao and colleagues [1, 2, 3]:

$$\text{Chao1} = S_{obs} + \frac{F_1(F_1-1)}{2(F_2+1)} \tag{1}$$

where S_{obs} is the observed number of species, and F_1 and F_2 are the numbers of singletons (only one count) and doubletons (exactly two counts), respectively, in each sample.
And for the ACE:

$$S_{ACE} = S_{abund} + \frac{S_{rare}}{C_{ACE}} + \frac{F_1}{C_{ACE}} + \gamma^2_{ACE} \tag{2}$$

where: S_{abund} and S_{rare} are the numbers of abundant and rare OTUs, with respect to a threshold of individuals in which OTUs are observed (3 in this study); C_{ACE} is the sample abundance coverage estimator obtained by $1 - \frac{F_1}{N_{rare}}$, with F_1 the frequency of singletons and $N_{rare} = \sum_{k=1}^{j} F_k$, for $j = 3$ the threshold for rare OTUs; γ^2_{ACE} is the coefficient of variation for OTU relative abundances:

$$\gamma^2_{ACE} = \max\left[\frac{S_{rare}}{C_{rare}} \cdot \frac{\sum_{k=1}^{j} k(1-k)F_k}{(N_{rare})(N_{rare}-1)} - 1, 0\right] \tag{3}$$

Diversity indexes : The Shannon index was obtained from [4]:

$$H' = -\sum_{i=1}^{S} (p_i \cdot ln(p_i)) \tag{4}$$

where p_i is the relative abundance of each OTU.
Similarly, the Simpson index was also based on OTUs relative abundances [5]:

$$D = 1 - \sum_{i=1}^{S} p_i^2 \tag{5}$$

Fisher's alpha [6] was estimated by solving numerically for alpha (Brent's algorithm [7] the following inverse Lambert-W function [8]:

$$S = \alpha \cdot \log\left(1 + \frac{N}{\alpha}\right) \tag{6}$$

where S is the number of OTUs and N is the number of individuals in the sample (log() is the natural logarithm).

Evenness indexes : Simpson's evenness measure E was calculated as:

$$E = \frac{1/D}{S_{obs}} \tag{7}$$

where D is the Simpsons's diversity from Equation 4 and S_{obs} is the observed number of species [9].
Pielou's J' index (a.k.a. Shannon's evenness) was obtained from the Shannon index (Equation 3) divided by the natural logarithm of the number of species [9, 10]:

$$J' = \frac{H'}{ln(S)} \tag{8}$$

Beta diversity

The across-sample rumen microbiota diversity was quantified by calculating Bray-Curtis dissimilarities [11]:

$$BC_{ij} = \frac{S_i + S_j - 2C_{ij}}{S_i + S_j} \tag{9}$$

Where S_i and S_j are the number of species in samples i and j, and C_{ij} is the number of species in common between the two samples (if the are no species in common, the numerator is equal to the denominator and the dissimilarity is one -maximum; if all species are in common, the dissimilarity is zero -minimum).

References

[1] Chao A. Nonparametric Estimation of the Number of Classes in a Population Author. Scanadinavian Journal of Statistics. 1984;11(4):265–270. doi:10.1214/aoms/1177729949.

[2] Chao A, Lee SM. Estimating the Number of Classes via Sample Coverage. Journal of the American Statistical Association. 1992;87(417):210. doi:10.2307/2290471.

[3] Chao A, Ma MC, Yang MCK. Stopping rule and estimation for recapture debugging with unequal detection rates. Biometrika. 1993;80(1):193–201. doi:10.1093/biomet/80.1.193.

[4] Shannon C. A Mathematical Theory of Communication, The Bell System Technical Journal. The Bell System Technical Journal. 1948; p. 379–427.

[5] Simpson EH. Measurement of diversity. Nature. 1949;163:688. doi:10.1038/163688a0.

[6] Fisher RA, Corbet AS, Williams CB. The Relation Between the Number of Species and the Number of Individuals in a Random Sample of an Animal Population. The Journal of Animal Ecology. 1943;12(1):42–58. doi:10.2307/1411.

[7] Brent RP. An algorithm with guaranteed convergence for finding a zero of a function. The Computer Journal. 1971;14(4):422–425. doi:10.1038/nmeth.f.303.

[8] Euler L. De serie Lambertina plurimisque eius insignibus proprietatibus. Acta Acad. Scient. Petropol. 1783;2:29–51. doi:10.1038/nmeth.f.303.

[9] Smith B, Wilson JB. A consumer's guide to evenness indices. Oikos. 1996;70–82.

[10] Pielou EC. Ecological diversity. John Wiley and Sons, New York. 1975.

[11] Bray JR, Curtis JT. An ordination of the upland forest communities of Southern Wisconsin. Ecol Monographs. 1957;27(4):325–349.
